# STING signaling activation modulates macrophage polarization via CCL2 in radiation-induced lung injury

**DOI:** 10.1186/s12967-023-04446-3

**Published:** 2023-09-04

**Authors:** Jianjiao Ni, Tiantian Guo, Yue Zhou, Shanshan Jiang, Long Zhang, Zhengfei Zhu

**Affiliations:** 1 Department of Radiation Oncology, Fudan University Shanghai Cancer Center, Shanghai, 200032 China; 2 Department of Oncology, Shanghai Medical College, Fudan University, 270 Dong An Road, Shanghai, 200032 China; 3 University of Shanghai for Science and Technology and Shanghai Changzheng Hospital Joint Research Center for Orthopedic Oncology, Institute of Biomedical Sciences and Clinical Technology Transformation, School of Health Science and Engineering, University of Shanghai for Science and Technology, 580 Jungong Road, Shanghai, 200093 China; 4 Institute of Thoracic Oncology, Fudan University, Shanghai, 200032 China

**Keywords:** Radiation-induced lung injury, cGAS-STING pathway, Macrophage polarization, Cytokine CCL2

## Abstract

**Background:**

Radiation-induced lung injury (RILI) is a prevalent complication of thoracic radiotherapy in cancer patients. A comprehensive understanding of the underlying mechanisms of RILI is essential for the development of effective prevention and treatment strategies.

**Methods:**

To investigate RILI, we utilized a mouse model that received 12.5 Gy whole-thoracic irradiation. The evaluation of RILI was performed using a combination of quantitative real-time polymerase chain reaction (qRT-PCR), enzyme-linked immunosorbent assay (ELISA), histology, western blot, immunohistochemistry, RNA sequencing, and flow cytometry. Additionally, we established a co-culture system consisting of macrophages, lung epithelial cells, and fibroblasts for in vitro studies. In this system, lung epithelial cells were irradiated with a dose of 4 Gy, and we employed STING knockout macrophages. Translational examinations were conducted to explore the relationship between STING expression in pre-radiotherapy lung tissues, dynamic changes in circulating CCL2, and the development of RILI.

**Results:**

Our findings revealed significant activation of the cGAS-STING pathway and M1 polarization of macrophages in the lungs of irradiated mice. In vitro studies demonstrated that the deficiency of cGAS-STING signaling led to impaired macrophage polarization and RILI. Through RNA sequencing, cytokine profiling, and rescue experiments using a CCL2 inhibitor called Bindarit, we identified the involvement of CCL2 in the regulation of macrophage polarization and the development of RILI. Moreover, translational investigations using patient samples collected before and after thoracic radiotherapy provided additional evidence supporting the association between cGAS-STING signaling activity, CCL2 upregulation, and the development of radiation pneumonitis.

**Conclusions:**

The cGAS-STING signaling pathway plays a crucial role in regulating the recruitment and polarization of macrophages, partly through CCL2, during the pathogenesis of RILI.

**Supplementary Information:**

The online version contains supplementary material available at 10.1186/s12967-023-04446-3.

## Introduction

Approximately 50–60% of cancer patients undergo radiotherapy either as a standalone treatment or in combination with surgery or chemotherapy to address their primary and metastatic malignancies [1]. One of the most common complications arising from thoracic radiation in cancer patients is radiation-induced lung injury (RILI). RILI was first identified in the early to mid-twentieth century when breast and lung cancer patients undergoing radiation treatment exhibited intrathoracic alterations [1]. Over the years, the etiology of radiation-induced lung damage has been extensively studied and characterized in clinical settings [1–3]. However, the molecular mechanisms underlying RILI are still not fully understood.

The administration of high-intensity radiotherapy is often limited due to the risk of lung tissue damage, which can manifest as radiation pneumonitis in approximately 30% of patients undergoing thoracic radiation [4, 5]. Radiation pneumonitis (RP) occurs as a result of an early inflammatory response triggered by damage to the lung parenchyma, epithelial cells, vascular endothelial cells, and stroma. This inflammatory process leads to the production of pro-inflammatory cytokines and chemokines that attract inflammatory immune cells to the lung tissue, ultimately causing pneumonitis and later fibrosis [6, 7]. Acute pneumonitis typically develops within 2–4 months after radiation treatment, while chronic fibrosis tends to present after 6 months following treatment. Unlike the normal wound healing process, late-stage radiation-induced pulmonary fibrosis is characterized by an abnormal resolution of inflammation [6].

cGMP-AMP (cGAMP) synthase (cGAS) is a cytosolic DNA sensor that specifically binds to double-stranded DNA (dsDNA) [8]. Upon binding to dsDNA, cGAS catalyzes the conversion of GTP and ATP into 2′3ʹ-cGAMP, which serves as a secondary messenger that activates the adaptor protein known as STING [8, 9]. Activated STING then recruits TANK-binding kinase 1 (TBK1) to initiate the activation of two key transcription factors: Interferon Regulatory Factor 3 (IRF3) and Nuclear Factor κB (NF-κB). IRF3 activation leads to the transcription of type I interferon (IFN-I) genes, while NF-κB stimulates the synthesis of inflammatory cytokines such as IL-1 and IL-6 [10]. The cGAS-cGAMP-STING pathway plays a crucial role in the immune defense against infections caused by various DNA-containing or DNA-generating viruses, as well as certain bacteria and parasites. Loss of cGAS or STING function significantly impairs the immune defense against these types of infections [8, 11, 12].

Radiotherapy not only directly targets and kills tumor cells, but also stimulates innate and adaptive immune responses through the STING-mediated DNA-sensing pathway. Deng et al. reported that STING-dependent cytosolic DNA sensing is essential for the induction of antitumor immunity by radiotherapy, primarily through modulation of type I IFN production [13]. Furthermore, Liang et al. demonstrated that radiotherapy-induced STING signaling promotes innate immune suppression by recruiting monocytic myeloid-derived suppressor cells into the tumor microenvironment through IFN production [14]. Nevertheless, the relationship between activated STING signaling by radiotherapy and the development of RILI is largely unknown. Further research is required to elucidate the specific mechanisms and implications of radiotherapy-induced STING signaling in the context of RILI.

In our study, we conducted observations in a mouse model of RILI and found notable activation of the cGAS-STING signaling pathway along with timing-dependent polarization of macrophages. Additionally, we conducted in vitro experiments where we confirmed the activity of STING signaling, assessed the population and polarization of macrophages, and evaluated the severity of RILI using the expression of well-recognized fibrotic proteins. Our findings demonstrated that the deficiency of cGAS-STING signaling resulted in impaired macrophage polarization and RILI, potentially through the regulation of the secretion of the cytokine CCL2. Furthermore, translational studies using patient’s samples collected before and after thoracic radiotherapy provided additional support to link the cGAS-STING signaling activity, CCL2 secretion and the development of RP. These results provide evidence for the involvement of the cGAS-STING pathway in regulating macrophage function and the development of RILI.

## Materials and methods

### Patients

We conducted a retrospective review of non-small cell lung cancer (NSCLC) patients who underwent curative surgery and subsequent thoracic radiation at Fudan University Shanghai Cancer Center (FUSCC) between January 2013 and December 2021. Eligible patients were required to have adequate specimens of normal lung tissues, defined as pulmonary tissues ≥ 5 cm away from the cancerous tissues in the postoperative lung samples stored at the Department of Pathology at FUSCC. Additionally, sufficient blood samples collected before and after thoracic radiotherapy, stored at the Department of Biobank at FUSCC, were also required. Exclusion criteria comprised a second primary tumor, neoadjuvant therapy, and post-radiotherapy follow-up of less than 6 months. The study adhered to the principles outlined in The Declaration of Helsinki and was approved by the institutional review board of FUSCC. Informed consent was waived by the institutional review board due to the retrospective nature of the study.

We collected common clinic-pathological parameters for each patient, including age, sex, comorbidities, smoking history, cancer stage, and histology. Additionally, information regarding thoracic radiation, including dose fractionation, start and finish dates, and concurrent systemic therapy, was also obtained. Surveillance contrast-enhanced computed tomography imaging was generally performed every 2–3 months, and the severity of RP was graded according to the Common Terminology Criteria for Adverse Events (CTCAE) version 5.0.

### Antibodies, drugs, and reagents

We utilized the following antibodies: STING (catalog 13647 for WB, 67733 for IHC, CST), pSTING (catalog 50907, CST), pTBK1 (catalog 5483, CST), TBK1 (catalog 38066, CST), pIRF3 (catalog 37829, CST), IRF3 (catalog 4302, CST), alpha-SMA (catalog A2547, Sigma-Aldrich), Collagen type I (ab292, Abcam), Vimentin (catalog ab92547, Abcam), and actin (catalog AC-40, Sigma-Aldrich). Bindarit (catalog SML2910) was purchased from Sigma-Aldrich.

### Cell culture and lentivirus generation

The RAW264.7 cell line and 293 T cells were cultured in Dulbecco’s Modified Eagle Medium supplemented with 10% fetal bovine serum, 100 units/mL penicillin, and 100 mg/mL streptomycin. Sf9 cells were grown in GIBCO® insect culture media supplemented with 10% fetal bovine serum, 100 units/mL penicillin, and 100 mg/mL streptomycin. Transfection of plasmid sgSTING in 293 T cells was performed using PEI (Polysciences) according to the manufacturer’s instructions. To generate lentivirus, 293 T cells were plated in 10-cm dishes and transfected with lentiviral plasmids and the lentiviral packaging plasmids pMD2.G and psPAX2 at a ratio of 1:0.5:1. After transfection for 48–72 h, the supernatant was collected using a 0.22 μm filter (Millipore, USA).

### Immunohistochemistry (IHC) stain and ELISA assay

Normal lung tissue specimens were stained using a primary rabbit anti-human STING antibody (ProteinTech Group, Wuhan, China) and scored as previously described [16]. Briefly, paraffin-embedded tissues were sectioned and stained according to the manufacturer’s instructions. The scoring system involved the multiplication of the intensity of immunohistochemical staining (0-no staining; 1-weak; 2-moderate; and 3-strong) and the percentage of positive tumor cells [1 (< 25%), 2 (25–50%), 3 (50–75%), 4 (> 75%)]. For each blood sample, a panel of circulating cytokines, including IL-2, IL-4, IL-10, IL-13, and CCL2, were measured using a Quantikine^®^ ELISA (enzyme-linked immunosorbent assay) kit (R&D Systems, Minneapolis, MN, USA) according to the manufacturer’s instructions.

### Animal treatments and irradiation procedure

C57BL/6 wild-type mice were purchased from Shanghai Silaike Experiment Animal Co., Ltd and acclimated for seven days before receiving further treatment. As previously described, the whole mice were exposed to a single dose of 12.5 Gy (0.5 Gy/min) of X-rays using a 60Co irradiator at the Department of Radiation Oncology, Fudan University Shanghai Cancer Center. Randomly, mice were divided into four groups (n = 21 per group): (1) the control (non-irradiation) + saline group (Saline), (2) the control (non-irradiation) + Bindarit group, (3) the irradiation + saline group (IR), and (4) the irradiation + Bindarit group. The mice were intratracheally administered saline (1 ml/kg) or Bindarit (50 mg/kg) one hour prior to the initial irradiation and sacrificed at 24 h, 48 h, one week, 4 weeks, 8 weeks, 12 weeks, and 24 weeks after radiation exposure, respectively.

### Flow cytometry

Macrophages were isolated from the lung tissues of the mice models using a macrophage isolation reagent (Peritoneum, Shanghai, China) to create the cell suspension. The surface was stained with CD11C (1 g/mL, 11-4714-42, Thermofisher) and CD206 (1 g/mL, 17-4801-82, Thermofisher), and the samples were analyzed using BD Fortessa. The results were analyzed using FlowJo (Tree Star, Ashland, OR) software.

### RNA extraction and Quantitative PCR analysis

Total RNA was extracted from lung tissues using TRIzol® (TaKaRa) and then reverse transcribed into cDNA using the PrimeScript RT-PCR Kit (TaKaRa, RR014) according to the manufacturer’s instructions. The cDNA products were subjected to quantitative PCR analysis using SYBR Mix (TaKaRa, RR064).

### Statistical analyses

Quantitative data are presented as mean ± SEM. An unpaired t-test was used to analyze differences between two groups. Multigroup differences were assessed by one-way ANOVA followed by the Tukey post hoc comparison test, using Prism 5 software. The statistical significance was set at P < 0.05.

## Results

### Thoracic radiation activated STING signaling and promoted RILI in mice

**Fig. 1:**
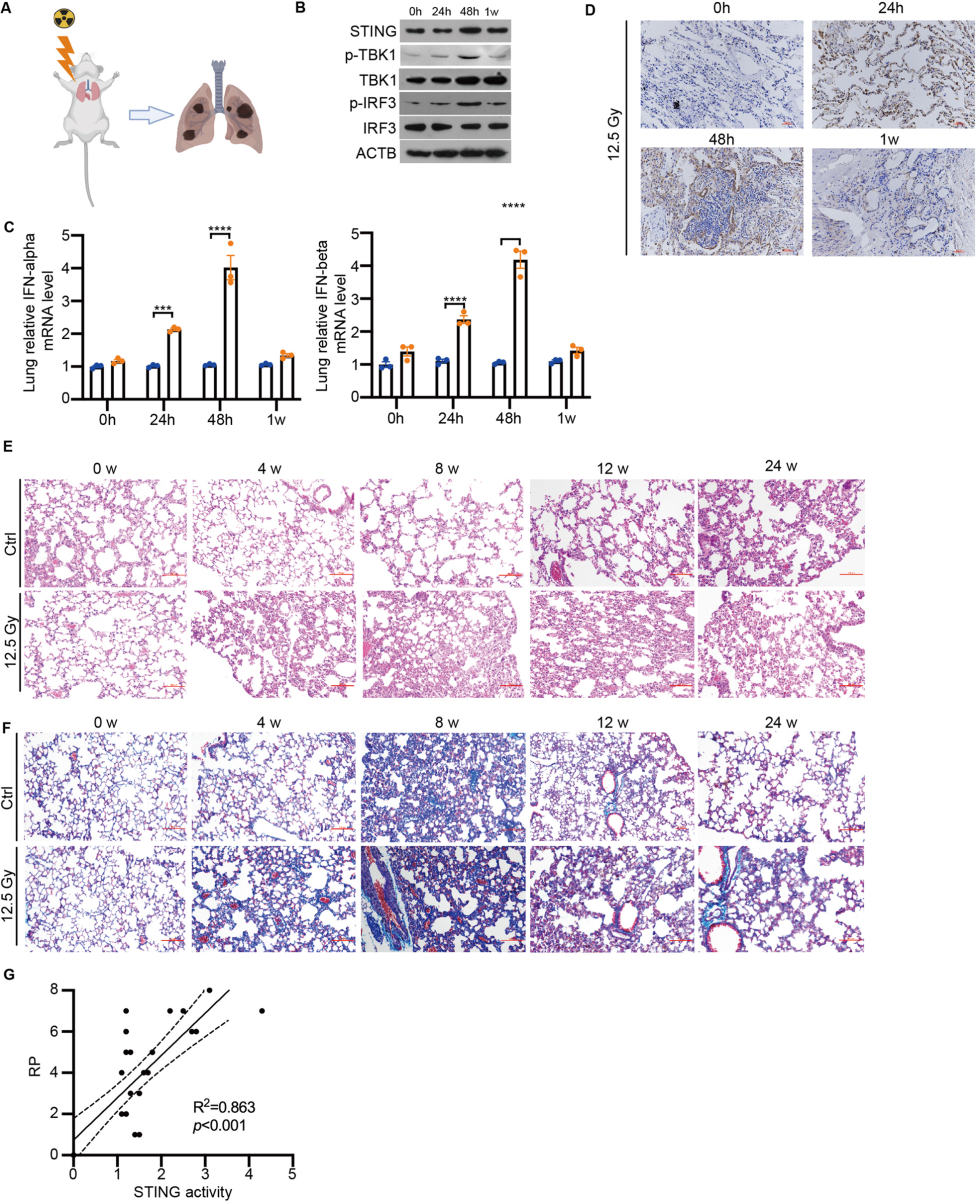
Thoracic radiation activated STING signaling and promoted RILI in mice. **A** A schematic diagram depicting the mice model of RILI is shown in A. **B** The proteins levels of STING signaling pathway in the lung tissues of RILI mice model. **C** Lung IFN-alpha and IFN-beta mRNA levels on 0 h, 24 h, 48 h and 1 week after irradiation in the RILI mice model (n = 3). **D** The phosphorylation levels of STING on 0 h, 24 h, 48 h and 1 week after irradiation in the lung tissues of RILI mice model(n = 6). **E** The H&E staining of lung tissues on 0, 4, 8, 12, 24 weeks after irradiation in RILI mice model(n = 6). Scale bars: 100 µm (20 × magnification). **F** The Masson`s trichrome staining of lung tissues on 0, 4, 8, 12, 24 weeks after irradiation in RILI mice model (n = 6). Scale bars: 100 µm (20 × magnification). **G** The correlation between the severity of RILI and the activity of the STING signaling pathway. Error bars represent mean ± SD. Statistical analyses were performed using two-tailed Students t-test or one-way ANOVA if more than two groups. ***P < 0.001; ****P < 0.0001

In order to investigate the impact of radiation on lung damage in mice and the potential activation of the STING signaling pathway, we established a mouse model of RILI by exposing the whole lung to a dose of 12.5 Gy (Fig. 1A). Through Western blot analysis, we observed a significant increase in the phosphorylation levels of TBK1 and IRF3, both downstream effectors of the STING signaling pathway, at 48 h post-irradiation. These levels generally returned to normal after 7 days (Fig. 1B). Consistent with the findings of Deng et al., irradiation also led to a significant increase in the mRNA expression levels of IFN-alpha and IFN-beta, which returned to baseline within one week (Fig. 1C). Additionally, we assessed the phosphorylation levels of STING in lung tissues and observed a significant upregulation at 24 and 48 h after irradiation, followed by a gradual normalization (Fig. 1D). Histological analysis using H&E staining revealed notable structural damage to the lungs of irradiated mice, characterized by edema, exudation, vascular congestion, thickening of the alveolar compartment, swelling of the alveolar interstitium, inflammatory infiltration, disintegration of alveolar walls, and lung consolidation (Fig. 1E). Masson staining further demonstrated the deposition of radiation-induced collagen around bronchi and vessels at 4 weeks post-irradiation (Fig. 1F). Moreover, we observed a correlation between the severity of RILI evaluated using a lung injury score [15] and the activity of the STING signaling pathway measured using the relative expression of IFN-Is (R^2^ = 0.863, P < 0.001), indicating a potential interaction between STING activation and the development of RILI (Fig. 1G). In summary, our findings suggest that thoracic radiation can activate the STING signaling pathway and induce lung injury in mice. These results provide insight into the potential involvement of STING signaling in RILI and its contribution to lung damage.

### Macrophages are polarized upon STING signaling activation in RILI mice model

**Fig. 2:**
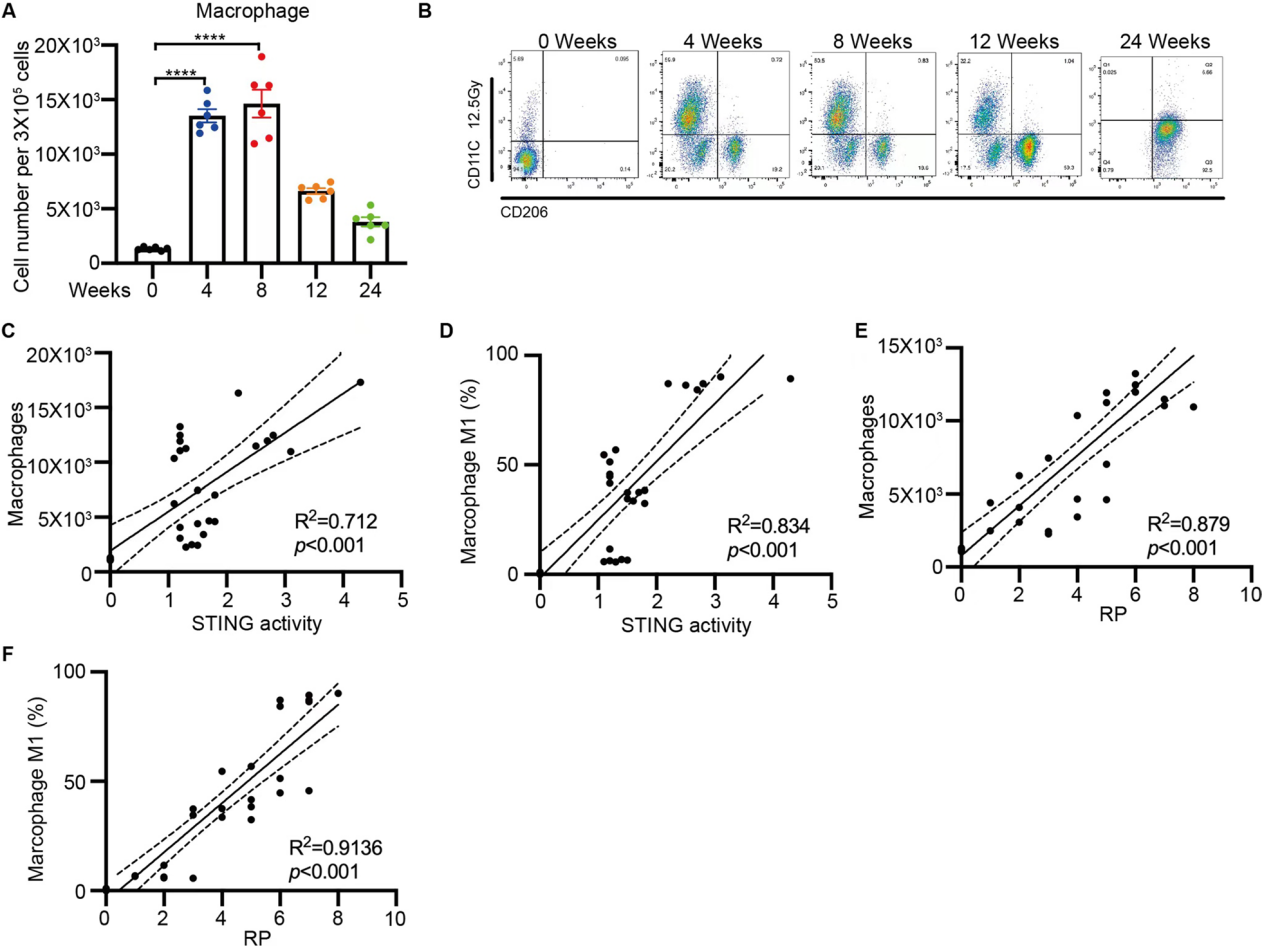
Macrophages are polarized upon STING signaling activation in RILI mice model. **A** The population of macrophages of lung tissues on 0, 4, 8, 12, 24 weeks after irradiation in RILI mice model(n = 6) **B** The macrophages polarization of lung tissues on 0, 4, 8, 12, 24 weeks after irradiation in RILI mice model by flow cytometry (n = 6). **C** The correlation between the population of macrophages and the activity of the STING signaling pathway. **D** The correlation between the macrophages M1 polarization percentage and the activity of the STING signaling pathway. **E** The correlation between the population of macrophage and the severity of RILI. **F** The correlation between the macrophages M1 polarization percentage and the severity of RILI. Error bars represent mean ± SD. Statistical analyses were performed using two-tailed Students t-test or one-way ANOVA if more than two groups. ****P < 0.0001

Accumulating evidence highlights the significant role of radiation in the recruitment and polarization of macrophages, which contribute to the establishment of a pro-inflammatory and pro-fibrotic environment [16, 17]. In our RILI model, we aimed to determine the status of macrophage enrichment and polarization by assessing their numbers and polarization markers in lung tissues. Following 4 weeks of irradiation, the number of macrophages increased from 1.7 × 10^3^ to 13.2 × 10^3^ per total 3 × 10^5^ cells and gradually declined after 12 weeks of irradiation (Fig. 2A). Additionally, we evaluated macrophage polarization in lung tissues using flow cytometry. CD11c is well-established as an M1 polarization marker [18] while CD206 is associated with M2 polarization [19]. Interestingly, we observed a significant increase in CD11c expression in macrophages from RILI mice during the first 8 weeks after irradiation. Conversely, CD206 expression showed an upward trend from 12 to 24 weeks post-irradiation (Fig. 2B). To explore the interplay between lung injury severity, STING activity, macrophage quantity, and macrophage polarization, we conducted pairwise correlation analyses. We discovered significant correlations between STING signaling activity and both the number of macrophages (R^2^ = 0.712, P < 0.001, Fig. 2C) and M1 polarization (R^2^ = 0.834, P < 0.001, Fig. 2D). Furthermore, strong positive associations were observed between the number of macrophages (R^2^ = 0.879, P < 0.001, Fig. 2E) or M1 polarization (R^2^ = 0.9136, P < 0.001, Fig. 2F) and the severity of RILI at 8 weeks after irradiation. Collectively, our findings indicate that thoracic radiation promotes the recruitment and polarization of macrophages in the lungs, which have been consistently implicated in the regulation of RILI. Moreover, these results suggest a potential interaction between the activation of the STING signaling pathway and the modulation of macrophages in the RILI model.

### STING deficiency impaired macrophage polarization and pulmonary fibrosis

**Fig. 3:**
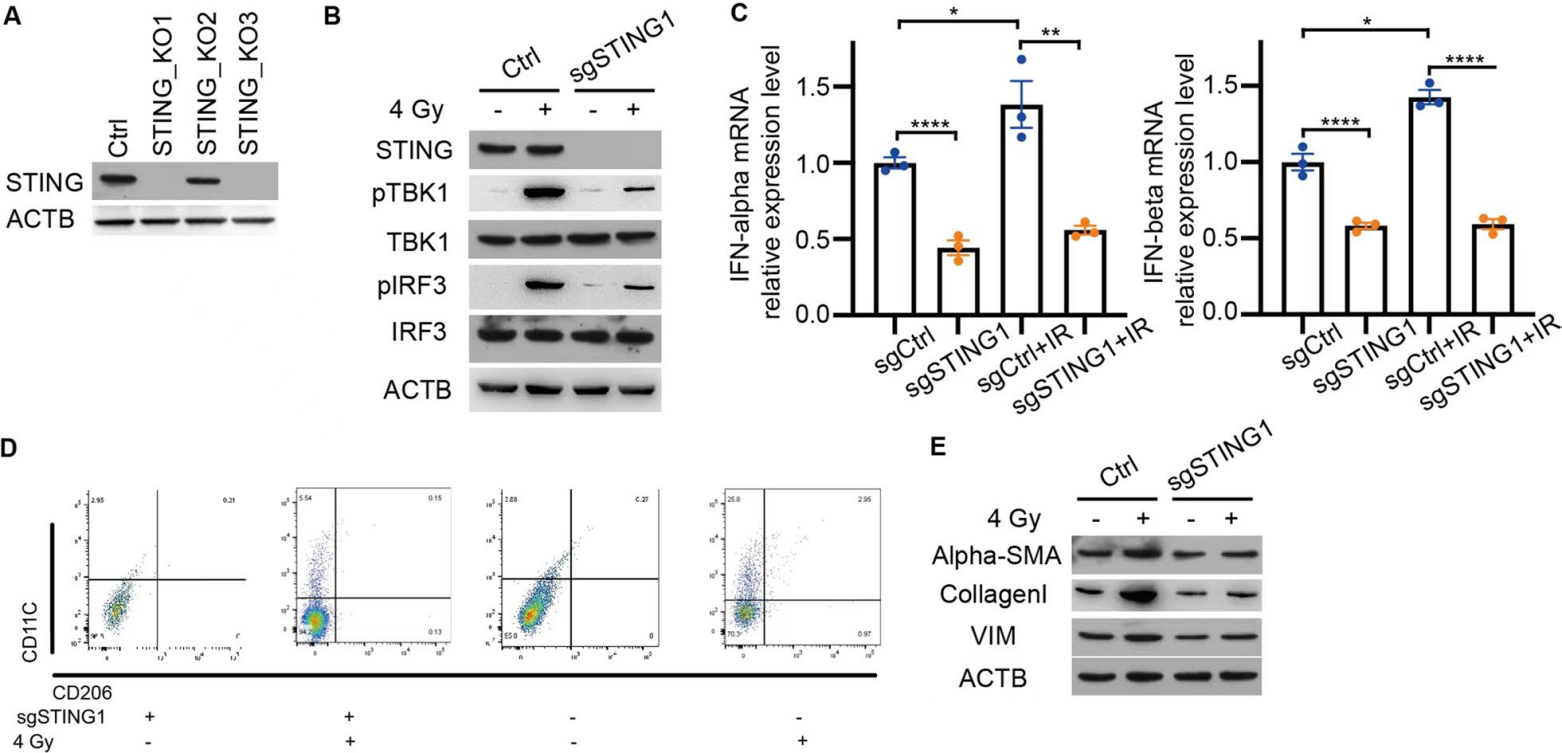
STING deficiency impaired macrophage polarization and pulmonary fibrosis. **A** The protein levels of STING after knocking out STING by CRISPR Cas9. **B** The proteins levels of STING signal pathway with or without STING in RAW264.7 cells. **C** The IFN-alpha and IFN-beta mRNA levels in RAW264.7 cells with or without STING after irradiation (n = 3). **D** The macrophages polarization of RAW264.7 cells with or without STING after irradiation (n = 3). **E** The protein levels of pulmonary fibrosis markers after irradiation in the co-culture system. Error bars represent mean ± SD. Statistical analyses were performed using two-tailed Students t-test or one-way ANOVA if more than two groups. *P < 0.05; **P < 0.01, ****P < 0.0001

To investigate the role of STING signaling in regulating macrophages in RILI, we established a co-culture system comprising macrophages, irradiated lung epithelial cells, and fibroblasts. We generated macrophages with or without STING expression using CRISPR/Cas9 technology. Three RAW264.7 cell lines with STING knockout were successfully generated, resulting in two cell lines with complete STING knockout (Fig. 3A). The absence of STING led to a significant decrease in the phosphorylation levels of TBK1 and IRF3 in macrophages when co-cultured with irradiated lung epithelial cells (Fig. 3B). Moreover, the mRNA levels of IFN-alpha and IFN-beta were diminished in STING-deficient macrophages (Fig. 3C). Notably, the proportion of macrophages exhibiting M1 polarization was reduced upon STING knockout during co-culture with irradiated lung epithelial cells (Fig. 3D). Furthermore, in the co-culture system, the production of alpha-SMA, collagen I, and Vimentin by fibroblast cells significantly increased when co-cultured with macrophages and irradiated lung epithelial cells. Excitingly, these markers lost their sensitivity to irradiation once STING was eliminated (Fig. 3E). These findings suggest that STING signaling plays a crucial role in regulating macrophage polarization, as well as the expression of collagen-related proteins in fibroblasts in the context of RILI. The absence of STING attenuates TBK1 and IRF3 phosphorylation, reduces IFN-I production, impairs macrophage polarization, and mitigates the fibrotic response in the co-culture system. These results highlight the significance of STING signaling in modulating the crosstalk between macrophages, lung epithelial cells, and fibroblasts during RILI.

### Changes of cytokines in RILI mice model

**Fig. 4:**
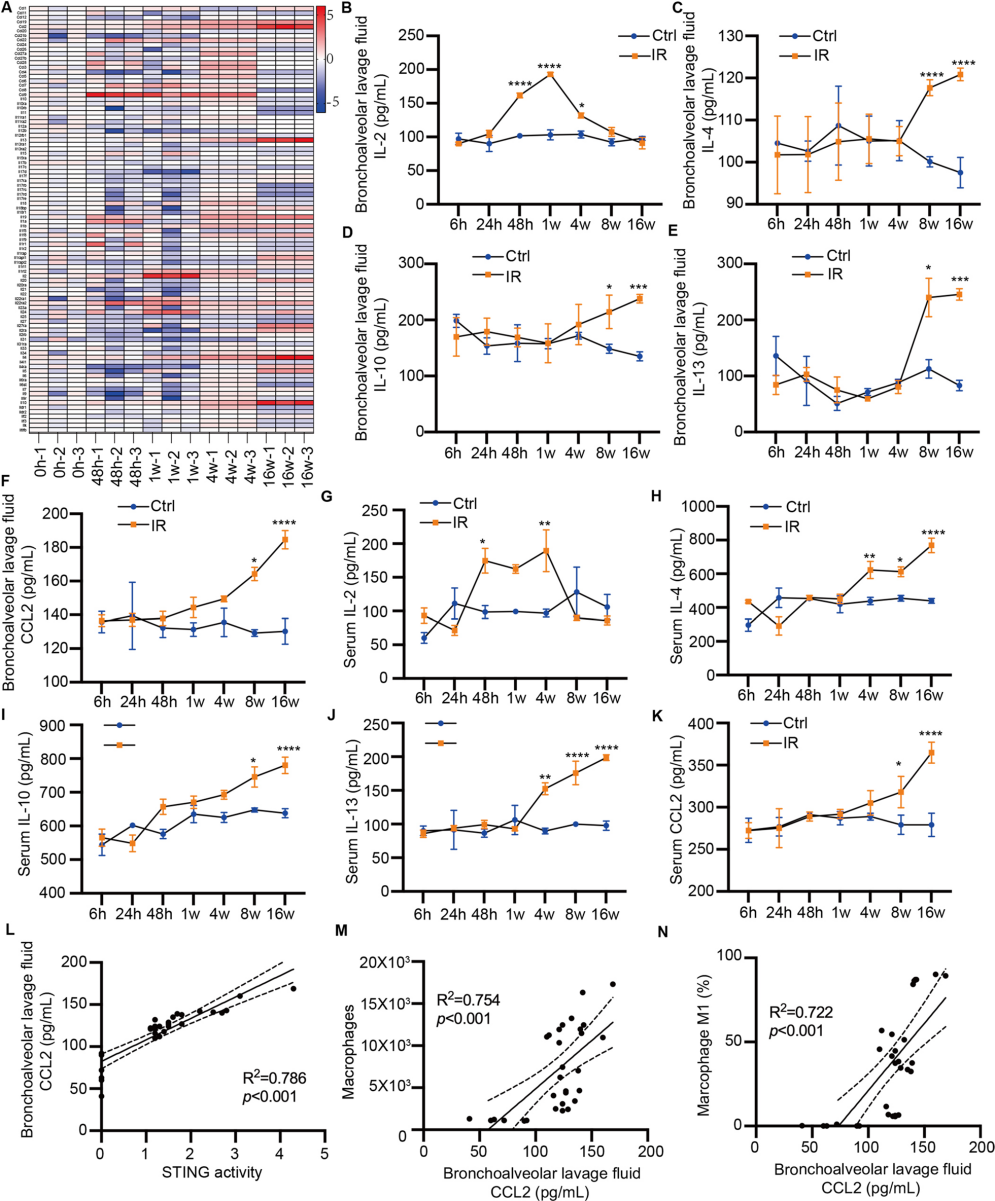
Changes of cytokines in RILI mice model. **A** The heatmap showing the cytokines mRNA levels by RNAseq. **B**–**F** The IL-2, IL-4, IL-10, IL-13 and CCL2 concentration in Bronchoalveolar lavage fluid 24, and 48 h after and 1, 4, 8, and 16 weeks after irradiation in RILI mice model by ELISA. **G**–**K** The IL-2, IL-4, IL-10, IL-13 and CCL2 concentration in serum 24, and 48 h after and 1, 4, 8, and 16 weeks after irradiation in RILI mice model by ELISA. **L** The correlation between the CCL2 concentration in Bronchoalveolar lavage fluid and the activity of the STING signaling pathway. **M** The correlation between the CCL2 concentration in Bronchoalveolar lavage fluid and the population of macrophages. **N** The correlation between the CCL2 concentration in Bronchoalveolar lavage fluid and the macrophages M1 polarization percentage. Error bars represent mean ± SD. Statistical analyses were performed using two-tailed Students t-test or one-way ANOVA if more than two groups. *P < 0.05; **P < 0.01; ***P < 0.001; ****P < 0.0001

To gain insights into the role of cytokines in the RILI mouse model, we performed RNA sequencing to analyze the dynamic changes of immune modulation proteins, including cytokines. Our results showed that certain cytokines, such as IL-1a, IL-2, and IL-19, exhibited a significant surge 48 h after irradiation, followed by a gradual decrease. On the other hand, cytokines like IL-4, IL-10, and IL-13 showed a gradual increase after weeks of irradiation (Fig. 4A). To validate the RNA sequencing data, we measured the concentrations of these cytokines in mouse bronchoalveolar fluids and sera using ELISA. We found that IL-2 levels in mouse bronchoalveolar fluids increased 24 h after irradiation, peaked at 1 week, and then returned to normal at 16 weeks (Fig. 4B). Interestingly, IL-4, IL-10 and IL-13 levels only rose at four weeks after irradiation (Fig. 4C–E). Similar patterns were observed in serum samples (Fig. 4G–J). Meanwhile, CCL2 levels began to rise 1 week after irradiation in mouse bronchoalveolar fluids (Fig. 4F) and 4 weeks after irradiation in mouse serum samples (Fig. 4K), respectively. Notably, CCL2 and its receptor, CCR2, are known to be involved in immune modulatory cell infiltration, particularly macrophages, in various disease settings. To investigate the relationship between CCL2 concentration and STING activity, macrophage number, and polarization, we analyzed their associations. We found that CCL2 concentration measured at 8 weeks after irradiation was positively correlated with STING activity (R^2^ = 0.786, P < 0.001, Fig. 4L), the number of macrophages (R^2^ = 0.754, P < 0.001, Fig. 4M), and M1 polarization (R^2^ = 0.722, P < 0.001, Fig. 4N). Taken together, our findings demonstrate that irradiation activates STING signaling and promotes the expression of different cytokines at distinct time points in the RILI model. Furthermore, CCL2 may be involved in the modulation of macrophage recruitment and polarization in RILI. These results provide valuable insights into the complex immune modulation and cytokine dynamics in the context of RILI.

### CCL2 antagonization reversed macrophage polarization and pulmonary fibrosis

**Fig. 5:**
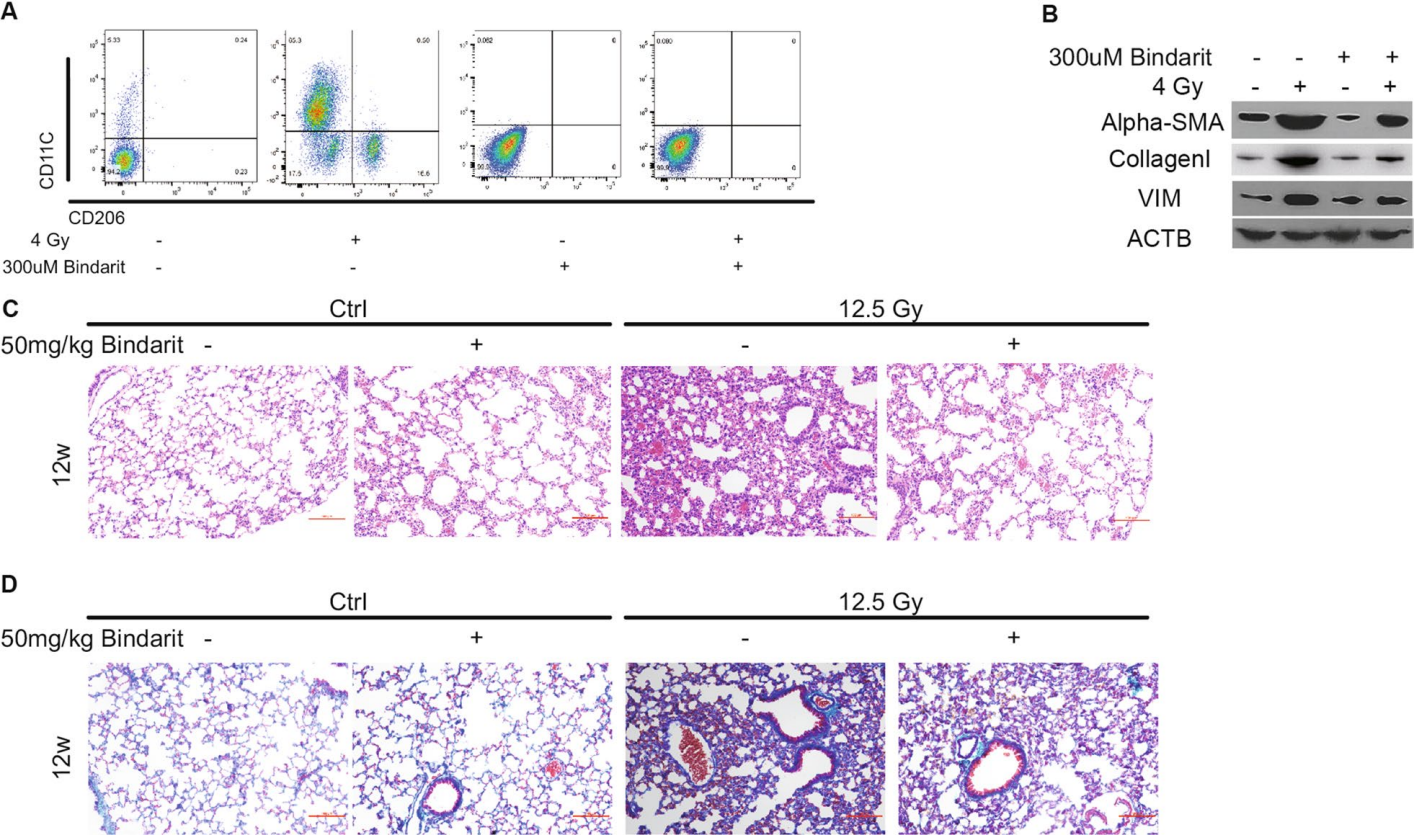
CCL2 antagonization reversed macrophage polarization and pulmonary fibrosis. **A** The macrophages polarization of lung tissues with or without Bindarit treatment on 0, 4, 8, 12, 24 weeks after irradiation in RILI mice model by flow cytometry (n = 6). **B** The protein levels of pulmonary fibrosis markers with or without Bindarit treatment in RILI mice model. **C** The H&E staining of lung tissues with or without Bindarit treatment in RILI mice model (n = 6). Scale bars: 100 µm (20 × magnification). **D** The Masson`s trichrome staining of lung tissues with or without Bindarit treatment in RILI mice model (n = 6). Scale bars: 100 µm (20 × magnification).

In our study, we further investigated the role of CCL2 in the recruitment and polarization of macrophages in the context of RILI. To assess the impact of CCL2 inhibition, we used the CCL2 inhibitor Bindarit in a co-culture system of macrophages, fibroblast, and lung epithelial cells, with or without irradiation. We found that treatment with 300 µM Bindarit significantly inhibited the M1 polarization of RAW264.7 cells after co-culture with irradiated lung epithelial cells (Fig. 5A). Moreover, Bindarit treatment at the same concentration led to a significant decrease in the protein levels of fibrosis indicators, including alpha-SMA, Collagen I, and Vimentin (Fig. 5B). To validate these findings in vivo, we administered Bindarit in mice models of RILI. Histological analysis using H&E and Masson stains revealed that Bindarit treatment alleviated RILI and pulmonary fibrosis in the mice models (Fig. 5C, D). Overall, our results indicate that CCL2 plays critical roles in the regulation of macrophage polarization and the development of pulmonary fibrosis. The use of the CCL2 inhibitor Bindarit demonstrated its potential as a therapeutic intervention to mitigate RILI and fibrosis. These findings suggest that targeting the CCL2 pathway could be a promising strategy for managing RILI-related complications.

### STING expression, dynamic changes of CCL2 and RILI in patients

**Fig. 6:**
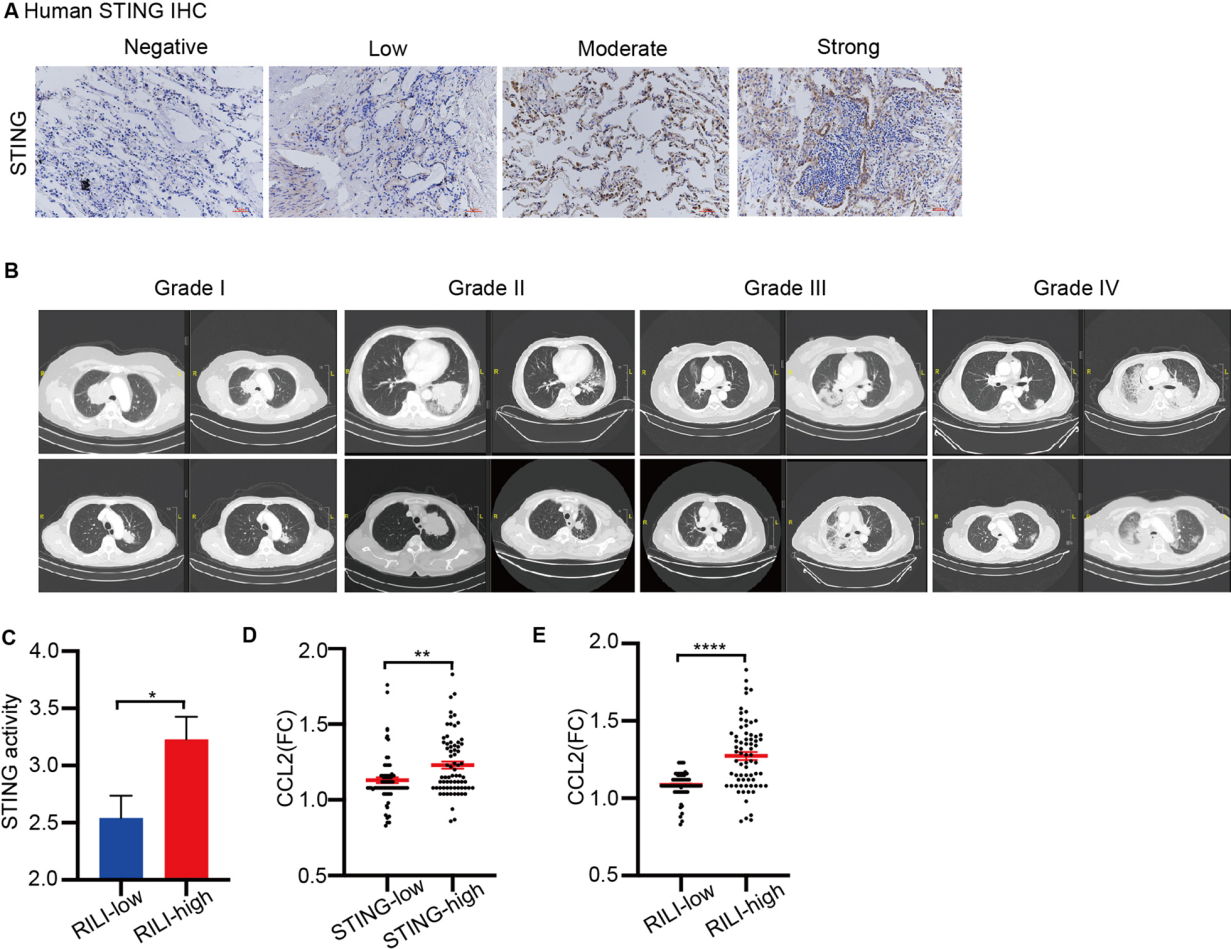
STING expression, dynamic changes of CCL2 and RILI in patients.** A** Representative images of STING expression in the normal lung tissue specimens by IHC. Scale bars: 100 µm (20 × magnification). **B** Representative CT scans from patients with various degrees of RILI. **C** The correlation between the severity of RILI and STING expression in the pre-radiotherapy lung tissues. **D** The correlation between fold changes of circulating CCL2 after radiotherapy and STING expression in the pre-radiotherapy lung tissues. **E** The correlation between the severity of RILI and fold changes of circulating CCL2 after radiotherapy. Error bars represent mean ± SD. Statistical significance was calculated using one way ANOVA by Tukey’s multiple comparisons test. *P < 0.05; **P < 0.01; ***P < 0.001

**Fig. 7:**
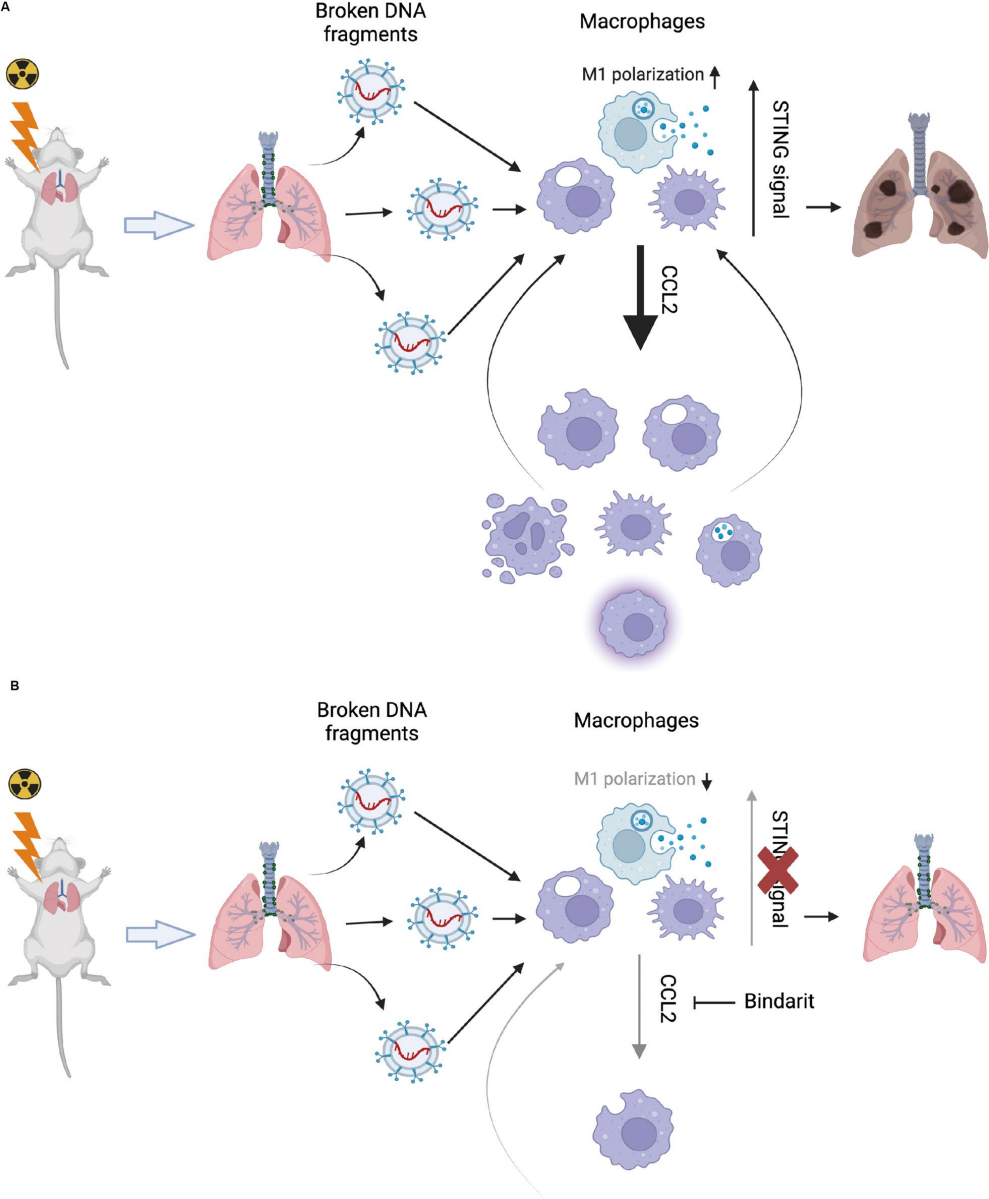
Schematic diagram of the mechanism of STING involved in RILI injury. **A** In RILI, the lung was damaged by radiation and emitted fragments of broken DNA. These fragments stimulated macrophage STING signaling activity. STING signaling activation promoted paracrine release of cytokines, such as CCL2, leading to the recruitment and polarization of macrophages in the lung, which eventually resulted in the development of RILI. **B** Deficiency in STING signaling, as well as the introduction of CCL2 inhibitor Bindarit, decreases the recruitment and polarization of macrophages, thereby alleviating RILI

In our clinical study involving 141 NSCLC patients receiving thoracic radiation (Additional file 1: Table S1), we aimed to validate the correlation between STING signaling, CCL2 secretion, and the development of RILI. We detected the expression levels of STING in pre-radiotherapy normal lung tissue samples from these patients (Fig. 6A). We found that there was no significant correlation between STING expression and common clinicopathological parameters in these patients. However, after radiation treatment, 29.1% of the patients developed symptomatic RP, with varying grades of severity (Fig. 6B). We observed a positive correlation between pre-radiotherapy STING expression and the development of symptomatic RP (Fig. 6C). Furthermore, we measured the expression levels of various circulating cytokines, including IL-2, IL-4, IL-10, IL-13, and CCL2, in pre-radiotherapy and post-radiotherapy plasma samples from these patients. Interestingly, we did not find a significant association between pre-radiotherapy STING expression and pre-radiotherapy circulating cytokines. However, higher pre-radiotherapy STING expression was associated with a significant upregulation of plasma CCL2 after thoracic radiotherapy (Fig. 6D). Importantly, the upregulation of plasma CCL2 after radiotherapy was correlated with the development of symptomatic RP in these patients (Fig. 6E). In conclusion, our clinical findings suggest that pre-radiotherapy STING expression in normal lung tissues, as well as the dynamic changes in circulating CCL2 levels after radiotherapy, could serve as important predictive biomarkers of symptomatic RP in patients undergoing thoracic radiation. These results provide valuable insights into the potential role of STING signaling and CCL2 in the pathogenesis of RILI and offer potential avenues for predictive and therapeutic strategies in the management of RILI (Fig. 7).

## Discussion

Our study has provided novel insights into the relationship between STING signaling, macrophages, and RILI. The activation of STING signaling in macrophages and their subsequent recruitment and polarization following irradiation play crucial roles in the pathophysiology of RILI. This finding is consistent with observations of radiation-induced changes in lung macrophages during different stages of tissue damage, indicating the contribution of macrophage activation to RILI.

The STING signaling pathway is known to be essential for the activation of innate immune responses against viral and bacterial infections. Macrophages, as part of the innate immune system, express STING and utilize this pathway to initiate anti-pathogen responses. Upon recognition of cytosolic DNA or cyclic dinucleotides, STING promotes downstream signaling cascades that lead to the production of type I interferons and other pro-inflammatory cytokines by macrophages. This activation of STING signaling in macrophages marks the initiation of an effective immune response against infections. Moreover, recent research has shown that the STING pathway may also regulate macrophage polarization and promote anti-tumor immunity, highlighting the intricate interaction between STING and macrophages in innate immunity [20, 21].

Numerous biomedical applications exist for the modulation of the STING signaling pathway. Firstly, activation of the STING pathway has been investigated as a novel therapeutic strategy for enhancing anti-viral or anti-tumor immune responses [22, 23]. Additionally, STING agonists have shown promise as vaccine adjuvants for the use in cancer and infectious disease vaccines [24, 25]. On other hand, inhibiting the STING pathway may be investigated as a means of suppressing the excessive immune responses observed in certain autoimmune and inflammatory conditions [26]. Finally, nanomaterials and their potential interactions with the STING signaling pathway have garnered a great deal of attention in biomedical research. Targeting cells and mitochondria in biomedical applications with green nanomaterials, also known as sustainable or eco-friendly nanomaterials, has emerged as a promising strategy [27, 28]. David R Wilson, et al. utilized biodegradable, poly(beta-amino ester) nanoparticles to deliver cyclic dinucleotides to the cytosol and induced a log-fold improvement in potency in treating established B16 melanoma tumors in vivo when combined with PD-1 blocking antibody in comparison to free cyclic dinucleotides without nanoparticles [29].

Given the crucial role of macrophages and the STING pathway in immune responses and tissue damage, it is not surprising that STING signaling is implicated in RILI. In radiation-induced liver disease, for example, the cGAS-STING pathway in non-parenchymal cells is rapidly activated by dsDNA released from hepatocytes, leading to the production and secretion of interferon-I and concurrent destruction of hepatocytes [30]. Similarly, single-cell sequencing of RILI mouse lung tissue has demonstrated the enrichment of the cGAS-STING signaling pathway and expansion of macrophages, further supporting the involvement of STING signaling in RILI [31]. Overall, our study has shed light on the intricate relationship between STING signaling, macrophages, and the development of RILI. By elucidating the molecular mechanisms underlying these interactions, the current study contributes to the understanding of the immune response to radiation-induced damage and may have implications for the development of therapeutic strategies targeting the STING pathway in RILI.

CCL2, also known as monocyte chemoattractant protein-1 (MCP-1), is a chemokine that regulates the recruitment and activation of immune cells. In response to inflammatory stimuli such as cytokines and bacterial or viral infections, a range of cell types, including macrophages, endothelial cells, and fibroblasts, generate CCL2 [32]. CCL2 stimulates the recruitment of monocytes and macrophages to areas of inflammation or damage by acting as a chemoattractant. Once recruited, macrophages may react to CCL2 by upregulating their expression of CCR2, the chemokine's receptor, and therefore enhancing their capacity to detect and respond to the chemokine. This establishes a positive feedback loop that increases macrophage recruitment and activation, resulting in a stronger immunological response. In addition, CCL2 may enhance the polarization of macrophages toward a pro-inflammatory phenotype, therefore boosting their capacity to phagocytose infections and generate cytokines [33]. Meanwhile, CCL2/CCR2 axis has also been implicated in the regulation of macrophage recruitment and polarization in the disease models of liver fibrosis [34, 35] and renal fibrosis [36]. In the current study, CCL2 expression was elevated just several days after irradiation and remained high throughout the whole process of RILI, which was consistent with the idea proposed by a previous study that CCL2 may be essential for controlling the recruitment and activation of macrophages throughout the whole immunological response [37].

Previous studies have examined the correlation between the concentration of various cytokines in peripheral blood and their dynamic changes and RILI, such as IL-6 [38], IL-8 [39], IL-10 [40] and TGF-beta [38]. We found that the CCL2 levels rose dramatically after irradiation and was linked with STING activity, which was demonstrated in our RILI mice models and partially supported in our translational data using pre- and post-radiotherapy blood samples from 141 NSCLC patients. In fact, CCL2 upregulation has been reported to be involved in the development of RILI [41–45]. Preliminary evidence from our study indicated potential mechanisms underlying the contribution of CCL2 upregulation in RILI through modulation of macrophages. Meanwhile, upregulation of CCL2 expression by the macrophages was linked to the STING signaling activation in the current study, which was in consistent with former findings in other disease models [46–49], with the exact mechanisms to be further elucidated.

The translational investigations of our study suggests that the expression of STING protein in the normal lung tissues and the dynamic changes of CCL2 in peripheral blood before and after radiotherapy have potential predictive values of RILI, which collaborated with a previous study suggesting significant association between dynamic changes of circulating CCL2 levels and symptomatic RP [50]. In the future, common clinicopathological parameters, changes in peripheral blood cytokines, and STING expression may be incorporated into a nomogram or prediction model that, following multi-center validation, can be used on a large scale.

## Conclusion

Activation of the cGAS-STING pathway regulates the polarization and function of macrophages, partially via CCL2, in the process of RILI. The expression of STING in the normal lung tissues, as well as the dynamic changes of circulating CCL2, could serve as predictive biomarkers of symptomatic RP in cancer patients receiving thoracic radiotherapy.

### Supplementary Information

**Additional file 1: Table S1.** Patient’s characteristics.

## Data Availability

The datasets used and/or analyzed during the current study are available from the corresponding author on reasonable request.

## Abbreviations

RILI: Radiation-induced lung injury
cGAS: CGMP-AMP (cGAMP) synthase
dsDNA: Double-stranded DNA
TBK1: TANK-binding kinase 1
IRF3: Interferon regulatory factor 3
NF-κB: Nuclear factor κB
IFN-I: Type I interferon
NSCLC: Non-small cell lung cancer
FUSCC: Fudan University Shanghai Cancer Center
RP: Radiation pneumonitis
CTCAE: The Common Terminology Criteria for Adverse Events
MCP-1: Monocyte chemoattractant protein-1
