# Supplementary material for: STING signaling activation modulates macrophage polarization via CCL2 in radiation-induced lung injury
Source: J Transl Med. 2023 Sep 4;21:590. doi: 10.1186/s12967-023-04446-3 (PMC10476398; doi:10.1186/s12967-023-04446-3)
Supplement: Supplementary file 1 — Additional file 1: Table S1. Patient’s characteristics. [file 12967_2023_4446_MOESM1_ESM.docx]

**Additional file Table S1.**  Patient’s characteristics

| **Baseline parameters** | **n** | **%** |
| --- | --- | --- |
| **Age at diagnosis (years)** |  |  |
| ≤65 | 76 | 53.9 |
| >65 | 65 | 46.1 |
| **Sex** |  |  |
| Female | 47 | 33.3 |
| Male | 94 | 66.7 |
| **Smoking history** |  |  |
| Ever smoker | 51 | 36.2 |
| Never smoker | 90 | 63.8 |
| **ECOG performance score** |  |  |
| 0 | 31 | 22.0 |
| 1 | 110 | 78.0 |
| **Histology** |  |  |
| Squamous cell carcinoma | 46 | 32.6 |
| Non-squamous NSCLC | 95 | 67.4 |
| **Clinical Stage** |  |  |
| II-III  IV  **Treatment**  Radiotherapy alone | 67  74  37 | 47.5  52.5  26.2 |
| Radiotherapy + systemic therapy | 104 | 73.8 |

ECOG, Eastern Corporative Oncology Group
